# A growing threat: Investigating the high incidence of benzimidazole fungicides resistance in Iranian *Botrytis cinerea* isolates

**DOI:** 10.1371/journal.pone.0294530

**Published:** 2023-11-21

**Authors:** Mohamad Mobasher Amini, Soheila Mirzaei, Ahmad Heidari

**Affiliations:** 1 Department of Plant Protection, Faculty of Agriculture, Bu-Ali Sina University, Hamedan, Iran; 2 Department of Pesticide Research, Iranian Research Institute of Plant Protection, Agricultural Research, Education and Extension Organization (AREEO), Tehran, Iran; Universitat Jaume 1, SPAIN

## Abstract

Effective management of fungicide application programs requires monitoring the profile of resistant populations of *Botrytis cinerea*, given its high-risk nature. This research aimed to examine the sensitivity of 200 *B*. *cinerea* isolates collected from different plant species and regions across Iran towards thiophanate-methyl and carbendazim fungicides. To distinguish between susceptible and resistant isolates, the discriminatory dose assay was employed, followed by the selection of representative isolates from each group for EC50 analysis. To identify potential modifications in codon 198 of the β-tubulin gene in *B*. *cinerea* resistant isolates, the researchers employed the PCR-RFLP diagnostic method. More than two-thirds of the isolates exhibited a varying degree of resistance to MBC fungicides, even in farms where the application of these fungicides had not taken place in recent years. After treatment with the *BsaI* enzyme, the PCR product of sensitive isolates displayed two bands measuring 98 and 371 bp, while only one band of 469 bp was identified in resistant isolates. The study also evaluated whether resistance to fungicides could affect the pathogenicity and mycelial growth of the isolates. The findings showed no significant difference between the resistant and sensitive groups in terms of these factors, indicating that resistance does not come at a cost to the pathogen’s fitness. Considering the high incidence of resistance and the absence of negative consequences on fitness, it is recommended to exercise caution in the employment of benzimidazole fungicides as part of *B*. *cinerea* management strategies.

## Introduction

*Botrytis cinerea* Pers.: Fr. is a ubiquitous and destructive phytopathogenic fungus that creates post and pre-harvest damages in more than one thousand horticultural, agricultural, and greenhouse-grown plant species like ornamental plants [1, 2]. This fungus has a necrotrophic lifestyle, is highly adaptable, and can survive in various climate conditions, with a variety of attack methods. It produces numerous conidia in host tissue and overwinters as spores, mycelia, and sclerotia in crop debris [3–5]. To mitigate the harmful effects of *B*. *cinerea* on plants, an effective management strategy is necessary. This strategy includes the use of fungicides, cultivating resistant plant varieties, implementing crop rotation, managing weeds, and adopting other cultural practices [6].

The use of fungicides has been severely impacted by the emergence of resistant isolates [7]. Multisite fungicides such as dithiocarbamates have been employed for a prolonged period to combat *B*. *cinerea*. Despite their non-specific mode of action, which results in a low risk of resistance development, there are several reports of reduced effectiveness of these fungicides against *B*. *cinerea*. Nowadays, single-site fungicide compounds are more prominent in chemical control strategies, and the use of multisite fungicides has diminished [8]. Site-specific fungicides are more effective than multisite compounds for controlling *B*. *cinerea*. Fungicides such as dicarboximide (DCFs), methyl benzimidazole carbamates (MBCs), quinone outside inhibitors (QoIs), anilinopyimidines (Aps), and succinate dehydrogenase inhibitors (SDHIs) are examples of site-specific fungicides that have been utilized against *B*. *cinerea* [5]. MBC fungicides, including carbendazim, fuberidazole, benomyl, thiabendazole, and thiophanate-methyl, were some of the earliest systemic single-site specific fungicides used to control *B*. *cinerea* in the 1970s [9]. These fungicides work by binding to the β-tubulin subunit and disrupting the fungal cytoskeleton, thus inhibiting germ tube elongation, mycelial growth, and nuclear division during mitosis [9, 10]. However, resistance to these fungicides emerged soon after their introduction [9]. Various studies have detected mutations at codons 198 and 200 in the β-tubulin gene that are accountable for conferring resistance to these fungicides. Specifically, amino acid substitutions of glutamic acid with valine (E198V), lysine (E198K), or alanine (E198A) in codon 198, and phenylalanine with tyrosine (F200Y) in codon 200 of the β-tubulin gene are associated with resistance [1, 7, 9, 11–13]. Therefore, it is essential to understand the resistance profile of *B*. *cinerea* populations in order to effectively manage the disease using fungicide applications.

The prevalence of *B*. *cinerea* in Iranian greenhouses, farms, and cold storages has made it a significant concern, leading to considerable amounts of annual spraying. Management strategies for addressing gray mold disease in Iran encompass several practices, including the removal and disposal of infected plant parts, pruning to enhance airflow, implementing proper agricultural techniques like plant spacing, and employing various approved pesticides as outlined by the Plant Protection Organization for gray mold management. Notably, the application of diverse fungicides stands as a crucial method for gray mold control in Iran. The regulation of pesticide use in Iranian agriculture dates back to 1966 when Iran’s Plant Protection Organization, the central authority overseeing agricultural pesticide management, enacted legislation mandating official registration for all agricultural pesticides [14]. Methyl thiophanate and carbendazim fungicides were first registered for use in Iran in 1971 and 1975, respectively, and were adopted as effective control agents for various fungal diseases [15]. A 2017 study by Morteza et al. [14] revealed the annual application of 170.4 tons of carbendazim and 112.3 tons of methyl thiophanate. While these fungicides were initially registered in Iran for diseases other than gray mold, their effectiveness in tackling this ailment in other countries led to their long-term use for gray mold control across various crops. Furthermore, although newer fungicides from different chemical groups like succinate dehydrogenase inhibitors (SDHIs) and quinone outside inhibitor (Q_o_I) have since been registered for *B*. *cinerea* disease control in Iran, benzimidazole fungicides continue to be utilized despite their disuse in other countries for gray mold control.

Nevertheless, studies on the resistance of this fungus to MBC fungicides in Iran have been limited [16]. To address this issue, the current study examined the resistance of 200 Iranian *B*. *cinerea* isolates to carbendazim and thiophanate-methyl.

## Results

### Effect of carbendazim and thiophanate-methyl on mycelial growth of *Botrytis cinerea*

The discriminatory concentration assay showed that the 200 *B*. *cinerea* strains could be categorized into three groups: sensitive, moderately resistant, and resistant. About 29% of the isolates were sensitive to carbendazim, with mycelial growth inhibition of over 90%. Meanwhile, 2.5% were moderately resistant, with mycelial growth inhibition between 20% and 90%, and 68.5% were resistant, with mycelial growth inhibition of less than 20%. For thiophanate-methyl, 29% of the isolates were sensitive, 2% were moderately resistant, and 69% were resistant. Based on the EC50 value, the isolates were further classified into three categories: sensitive (EC50 < 1 μg/ml), low resistant (1 < EC50 < 10 μg/ml), and highly resistant (EC50 > 100 μg/ml) to thiophanate-methyl, and two categories (sensitive and highly resistant) for carbendazim. Tables 1 and 2 provide the EC50 values for different isolates.

**Table 1:** The result of EC50 value test in carbendazim fungicide.

Isolate name	Sampling region (province)	Host	GI (%)	State of resistance and sensitivity based on discriminatory concentration	EC50 (μg/ml)	State of resistance and sensitivity based on EC50
AH1	Hamedan	tomato	100	S	0.012	S
AH5	Hamedan	grape	100	S	0.279	S
AKA11	Alborz	rose	100	S	0.092	S
AH26	Hamedan	aloe vera	100	S	0.154	S
AH90	Hamedan	tomato	100	S	0.219	S
AH93	Hamedan	tomato	100	S	0.138	S
AH124	Hamedan	tomato	100	S	0.090	S
AT1	East Azarbaijan	peach	100	S	0.216	S
AK25	Kordestan	strawberry	100	S	0.092	S
AKA2	Alborz	gundelia	100	S	0.138	S
AH4	Hamedan	cucumber	11.94	R	>800	HR
AH24	Hamedan	cineraria	0.62	R	>800	HR
AH28	Hamedan	cucumber	28.78	MR	>800	HR
AH41	Hamedan	tomato	1.36	R	>800	HR
AH67	Hamedan	tomato	0.92	R	>800	HR
AH85	Hamedan	tomato	1.29	R	>800	HR
AH91	Hamedan	tomato	0.64	R	>800	HR
AH98	Hamedan	tomato	1.44	R	>800	HR
AH104	Hamedan	tomato	53.15	MR	>800	HR
AH119	Hamedan	tomato	0	R	>800	HR
AH122	Hamedan	tomato	0.79	R	>800	HR
AH132	Hamedan	tomato	2.75	R	>800	HR
AH133	Hamedan	tomato	0	R	>800	HR
AZ3	Zanjan	strawberry	5.65	R	>800	HR
AK15	Kordestan	strawberry	2.08	R	>800	HR
AK21	Kordestan	strawberry	8.35	R	>800	HR
ATE2	Tehran	basil	15.69	R	>800	HR
ATE3	Tehran	lettuce	6.97	R	>800	HR
AKA8	Alborz	cauliflower	1.96	R	>800	HR
AH94	Hamedan	tomato	7	R	>800	HR

GI: mycelial growth inhibition in discriminate concentration, EC50: the effective concentration that reduces 50% of mycelial growth, R: resistant, S: sensitive, MR: moderately resistant, HR: highly resistant, LR: low-resistant.

**Table 2:** The result of the EC50 value test in thiophanate-methyl fungicide.

Isolate name	Sampling regions (province)	Host	GI (%)	State of resistance and sensitivity based on discriminatory concentration	EC50 (μg/ml)	State of resistance and sensitivity based on EC50
AH39	Hamedan	tomato	100	S	1.30	LR
AH25	Hamedan	impatiens	100	S	1.31	LR
AH42	Hamedan	grape	100	S	1.03	LR
AH57	Hamedan	tomato	100	S	0.961	S
AH128	Hamedan	tomato	100	S	1.68	LR
AK3	Kordestan	strawberry	100	S	1.521	LR
AK9	Kordestan	strawberry	100	S	1.563	LR
AKA2	Alborz	gundelia	100	S	0.948	S
AKA11	Alborz	rose	100	S	1.409	LR
AT1	East Azarbaijan	peach	100	S	1.559	LR
AH2	Hamedan	tomato	2.09	R	>1280	HR
AH4	Hamedan	cucumber	0.56	R	487.06	HR
AH14	Hamedan	tomato	7.97	R	>1280	HR
AH24	Hamedan	cineraria	1.12	R	>1280	HR
AH28	Hamedan	cucumber	14.78	R	418.9	HR
AH35	Hamedan	tomato	09.06	R	728.1	HR
AH44	Hamedan	strawberry	11.49	R	974.9	HR
AH66	Hamedan	tomato	14.83	R	260.8	HR
AH69	Hamedan	tomato	6.64	R	>1280	HR
AH85	Hamedan	tomato	8.59	R	>1280	HR
AH100	Hamedan	tomato	3	R	>1280	HR
AH101	Hamedan	tomato	6.52	R	>1280	HR
AH104	Hamedan	tomato	53.18	MR	1174.8	HR
AH119	Hamedan	tomato	1.07	R	1030.6	HR
AH132	Hamedan	tomato	6.04	R	>1280	HR
AH145	Hamedan	tomato	9.5	R	1170.9	HR
AZ3	Zanjan	strawberry	0	R	>1280	HR
AK1	Kordestan	strawberry	3.46	R	>1280	HR
ATE2	Tehran	basil	12.44	R	>1280	HR
ATE3	Tehran	lettuce	1.38	R	1021.3	HR

GI: mycelial growth inhibition in discriminate concentration, EC50: the effective concentration that reduces 50% of mycelial growth, R: resistant, S: sensitive, MR: moderately resistant, HR: highly resistant, LR: low-resistant.

### Fitness of resistant and sensitive strains

There was no significant difference between resistant and sensitive groups in terms of mycelial growth or pathogenicity. In both groups, some isolates were pathogenic, while others were not (Table 3). These observations suggest that resistance was not linked to any adverse effect on fitness.

**Table 3:** Mean comparison of mycelial growth and pathogenicity as fitness parameters.

carbendazim				thiophanate-methyl			
Isolate name	Sensitivity (EC50)	Mycelial growth*	Pathogenicity	Isolate name	Sensitivity (EC50)	Mycelial growth	Pathogenicity
AH104	HR	3.97^e^±0.252	1.98^a^±0.076	AH104	HR	5.17^c^±0.231	1.98^a^±0.076
AH85	HR	5.13^bc^±0.115	1.58^ab^±0.388	AT1	LR(S)**	5.83^a^±0.058	1.30^ab^±0.265
AH132	HR	4.80^bcd^±0.1	1.32^ab^±0.076	AH100	HR	5.53^b^±0.058	1.25^ab^±0.18
ATE2	HR	4.7^d^±0.058	1.22^ab^±0.076	AH44	HR	5.50^b^±0.1	1.20^b^±0.1
AH26	S	3.77^e^±0.289	1.20^ab^±0.1	ATE3	HR	4.97^cd^±0.153	1.10^b^±0.1
AH90	S	5.10^bcd^±0.1	1.12^b^±0.161	AH4	HR	5.90^a^±0.00	1.10^b^±0.1
AH1	S	5.77^a^±0.153	1.10^b^±0.265	AH42	LR(S)	5.83^a^±0.058	1.03^b^±0.058
AH124	S	5.23^b^±0.058	1.03^b^±0.058	AKA2	S(S)	5.50^b^±0.1	0
AKA11	S	3.57^e^±0.115	0	AK9	LR(S)	4.63^d^±0.058	0
AK21	HR	4.73^cd^±0.321	0	AH39	LR(S)	5.83^a^±0.058	0

R: resistant, S: sensitive, HR: highly resistant, LR: low-resistant

*Mean of values ± standard deviation of the respective traits, values followed by different letters are significantly different according to a Duncan test at p < 0.01

**(S): according to discriminatory concentration

### Molecular analysis of β-tubulin gene by PCR-RFLP assay

**Fig 1:**
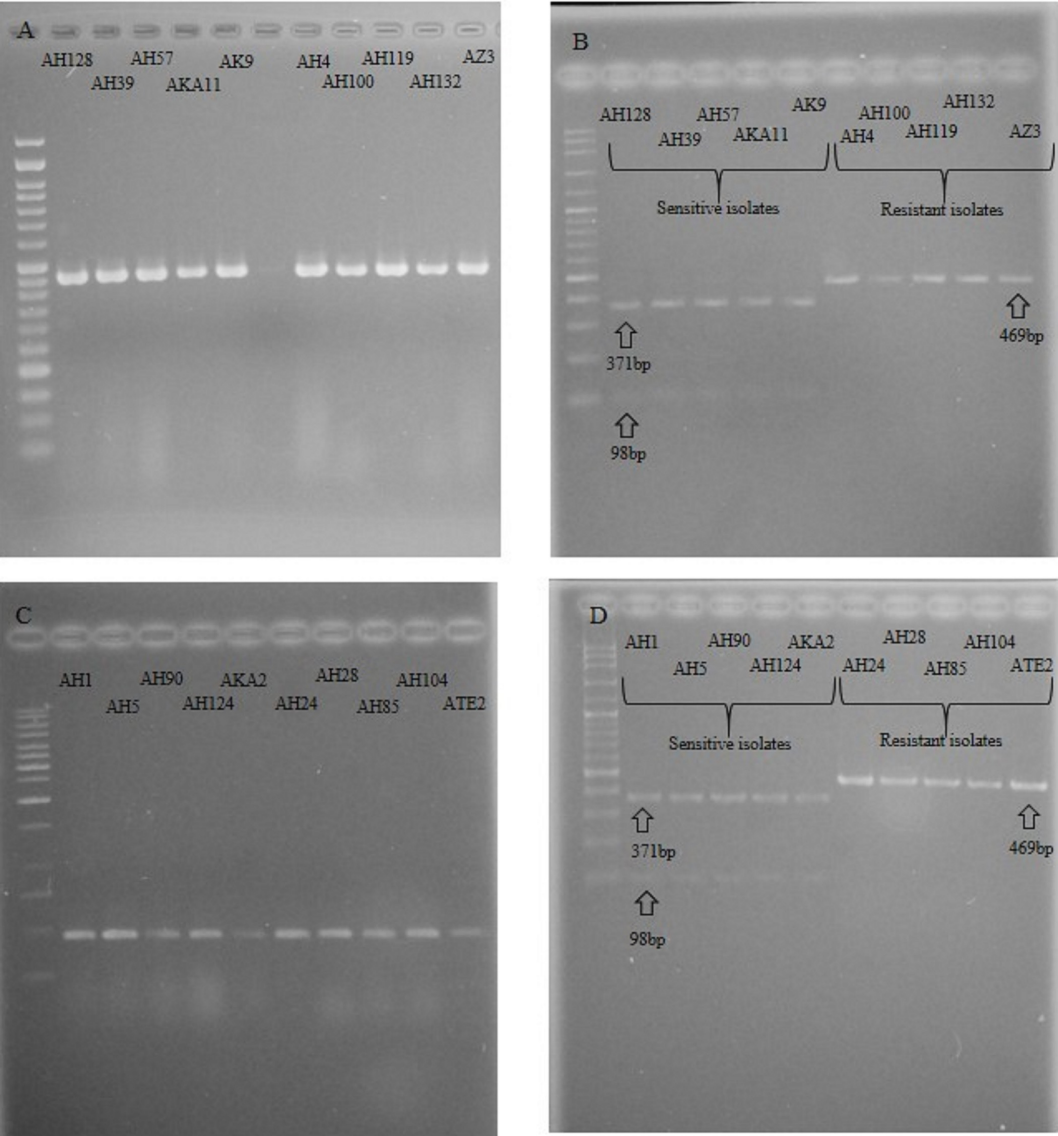
PCR-RFLP detection of *Botrytis cinerea β*-tubulin gene mutations. A: Amplified PCR products of the β-tubulin gene in sensitive and resistant isolates to thiophanate-methyl, B: *BsaI* digestion of the β-tubulin gene PCR-products in thiophanate-methyl resistant and sensitive isolates. C: Amplified PCR products of the β-tubulin gene in sensitive and resistant isolates to carbendazim, D: *BsaI* digestion of the β-tubulin gene in carbendazim resistant and sensitive isolates.

In this study, a PCR-RFLP diagnostic method was utilized to investigate the potential modifications in codon 198 of the β-tubulin gene in *B*. *cinerea* resistant isolates. The method involved the use of the *BsaI* restriction enzyme which recognizes the restriction site at amino acids 196–199 in *B*. *cinerea* sensitive isolates but not in resistant isolates due to certain mutations such as E198G, E198V, E198A, and E198K. Upon digestion of the PCR product from sensitive isolates with *BsaI* enzyme, two bands of 98 and 371 bp were observed, whereas only one band of 469 bp was detected in resistant isolates (Fig 1). The findings were in consistent with the results of the EC50 assay.

## Discussion

This study aimed to investigate the susceptibility of 200 *B*. *cinerea* isolates obtained from various plants in diverse regions of Iran to thiophanate-methyl and carbendazim fungicides. The discriminatory dose assay was used to differentiate between sensitive and resistant isolates, after which 30 representative isolates from each group were selected for EC50 analysis. The selected isolates exhibited EC50 values ranging from 0.012 to over 800 ppm for carbendazim and 0.948 to over 1280 ppm for thiophanate-methyl. Previous studies have also reported the existence of isolates with high EC50 values that can withstand high fungicide concentrations [6, 10, 17, 18].

The mutation in the β-tubulin gene has been found to be responsible for resistance to MBC fungicides, and the effects of this mutation have been examined by several researchers [11, 16, 19, 20]. In the current study, the PCR-RFLP method was employed to analyze the resistant and sensitive *B*. *cinerea* isolates. The band pattern observed in electrophoresis of PCR products was consistent with that of resistant and sensitive isolates, which is in line with previous findings [7, 12]. Different levels of resistance were found in the isolates towards carbendazim and thiophanate-methyl. Avenot et al. [10] reported that mutations in β-tubulin gene regions associated with high levels of *Botrytis* resistance to thiophanate-methyl were not related to low levels of resistance. In the present study, isolates with low levels of resistance to thiophanate-methyl in the EC50 test exhibited the pattern of sensitive isolates in PCR-RFLP. Therefore, it is likely that mutations in other parts of the β-tubulin gene are responsible for low levels of resistance.

The overuse of fungicides coupled with the high genetic variability of *Botrytis cinerea* has led to the emergence of resistant isolates. In Iran, due to indiscriminate fungicide use by some farmers, the appearance of resistant isolates is a concern. Rezaee et al. [16] found that 38% of *B*. *cinerea* isolates obtained from grapes in the West Azarbaijan province of Iran were resistant to benzimidazole. The present study shows that over two-thirds of isolates had some degree of resistance to MBC fungicides, including some that were found in farms where these fungicides had not been used in recent years. Walker et al. [21] showed that resistance to carbendazim persisted in the population even many years after limited use. Other studies have also found high levels of resistance to MBCs, with Lopes et al. [4] reporting 93% of isolates resistant to thiophanate-methyl.

In two consecutive years of sampling in four greenhouses, the majority of the collected isolates showed resistance towards carbendazim and thiophanate-methyl. In one greenhouse, all isolates were resistant, while in the other greenhouse, more than 70% of the isolates were resistant in both years. In the third greenhouse, half of the collected isolates were sensitive in the first year, but all isolates showed resistance in the following year. The use of only carbendazim in this greenhouse was the likely cause of resistance to thiophanate-methyl as well since they have the same mode of action. A greenhouse where all isolates were initially resistant showed some sensitive isolates in the second year, probably due to the movement of sensitive isolates from other greenhouses. Pathogenicity and growth rate studies showed no significant difference between resistant and sensitive isolates, indicating no fitness cost for resistance.

The sub-population analysis revealed that *vacuma* and *transposa* had a higher proportion of resistant isolates than other sub-populations. However, due to the small sample size in these sub-populations, a definitive conclusion cannot be drawn.

## Conclusion

In conclusion, the study found a high percentage of resistance to carbendazim and thiophanate-methyl in 200 isolates of *B*. *cinerea* obtained from various hosts and regions in Iran. The resistance was caused by a mutation in the β-tubulin gene as detected by the PCR-RFLP method. Fitness assays revealed no significant association between resistance and fitness cost. Due to the high prevalence of resistance and lack of fitness penalty, caution must be taken in the use of MBC fungicides in management strategies. Combining other management methods with fungicide use and monitoring resistance over time can help reduce the risk of increasing fungicide-resistant populations.

## Materials and methods

### Fungal isolates

Between 2015 and 2021, a total of 163 *B*. *cinerea* isolates were obtained from various hosts, with an additional 37 isolates provided by other researchers. These 200 isolates were obtained from multiple provinces throughout Iran, sourced from 26 distinct hosts, including but not limited to tomato, strawberry, and rose, and collected during various seasons. The sampling process was conducted in areas with different weather conditions and latitudes, with the fungicide use history being recorded where available. Four greenhouses were sampled over two years, where previous fungicide use was known. The isolates were purified through single spore and hyphal tip techniques, then kept on PDA (potato dextrose agar) at 4°C or under glycerol (%20) at -20°C. Among the 200 isolates collected from various hosts and regions in Iran, 35 were analyzed in a previous study for transposons (unpublished data). Of these, 17 were *transposa*, 7 were *vacuma*, 9 were *flipper*, and 2 were *boty*.

### Fungicide sensitivity assay

The research utilized two fungicides, namely thiophanate-methyl (70% WP, Insecticides India LTD., India) and carbendazim (98.6% TC, Shandong Weifang Rainbow Chemical Co., LTD, China), to assess their sensitivity and resistance through the agar plates method as explained by Plesken et al [3]. The discriminatory doses of 5 ppm for carbendazim and 10 ppm for thiophanate-methyl were used to distinguish resistant and sensitive isolates. Thirty isolates out of 200 were chosen, representing various categories (sensitive, moderately resistant, and resistant), to determine the effective concentration that reduces 50% of mycelial development (EC50). As the resistance profiles of all 200 isolates were identical for both fungicides (as anticipated, given their shared target site), we opted to individually select isolates to diversify our sample for subsequent investigations and each fungicide’s selection process was done separately. Nevertheless, it’s important to note that among the isolates selected for each fungicide, there were 13 isolates that were common to both sets of selections.

The EC50 values were assessed for the representative isolates using a range of concentrations. For carbendazim, the concentrations were 0, 0.1, 0.5, 1, 2, 4, 6, 100, 200, 400, and 800 mg/L active ingredient. For thiophanate-methyl, the concentrations were 0, 0.1, 1, 2, 4, 8, 10, 16, 80, 160, 320, 640, and 1280 mg/L active ingredient. To prepare the stock solution, the fungicides were dissolved in sterile water, and aliquots of the solution were added to autoclaved culture media (PDA) at 40°C to achieve the required concentrations. Mycelial plugs were taken from the edge of a 4-day-old colony and placed onto PDA plates with and without fungicide. The colony diameter was measured after a 4-day incubation in darkness at 22°C, and the percentage of mycelial growth inhibition was calculated for each isolate. The *B*. *cinerea* isolates were then categorized as highly resistant (EC50 > 100 μg/ml), moderately resistant (50 < EC50 < 100 μg/ml), weakly resistant (10 < EC50 < 50 μg/ml), low resistant (1 < EC50 < 10 μg/ml), and sensitive (EC50 < 1 μg/ml) based on the calculated EC50 values [10].

### Evaluation of fitness parameters

Ten isolates, comprising of both sensitive and resistant strains, were chosen to investigate if resistance comes with a cost to the fitness of the pathogen. For each fungicide, the selection process was conducted independently, and the fitness was assessed based on pathogenicity and mycelial growth of the isolates. Mycelial growth was determined by transferring 5 mm diameter mycelial plugs from the edge of the growing culture to the center of PDA-containing Petri dishes without fungicides. After incubating in the dark at 22°C for four days, the colony diameter was measured. To evaluate pathogenicity, mycelial plugs of 5 mm diameter obtained from three-day-old colonies were placed on small scratches created on the surface of sterilized grape leaves using a needle. The leaves were then incubated in the dark at 25°C, and the lesion diameter was measured four days post-inoculation.

### Data analysis

The study was carried out following a completely randomized design with three replications, and the statistical analysis was conducted using analysis of variance (ANOVA). Significant differences between means were compared using the Duncan test with a significance level of 1% (alpha = 0.01). The statistical software SAS 9.1 was employed to perform the analysis. The EC50 value was obtained by performing a linear regression of mycelial growth inhibition against the log10-transformed concentrations. To determine the normality of the data, the Kolmogorov-Smirnov test was used, and when necessary, the Cox-box method was applied to normalize the data. SPSS ver.26.0.0.0 (IBM SPSS Statistics, USA) was used for data analysis.

### Molecular study

#### DNA extraction

Ten isolates that represented both resistant and sensitive phenotypes were selected for molecular investigation. The isolates were grown on 10 cm Petri dishes containing PDA and incubated at 22°C for 4 days in darkness. Mycelium was collected from the surface of PDA using a sterile scalpel, and DNA was extracted using the method described by Moller et al. [22].

#### Molecular analysis of β-tubulin by PCR-RFLP assay

The gene of interest, β-tubulin, which is the target for the fungicides thiophanate-methyl and carbendazim, was amplified using the primer pairs bcF: 5′- GGCTACCTTCTCCGTCGTC-3′ and bctubR: 5′- AAAATGGCAGAGCATGTCAA -3′. The PCR protocol involved an initial step of 15 minutes at 95°C, followed by 35 cycles of 30 seconds at 94°C, 30 seconds at 55°C, and 30 seconds at 72°C, with a final extension of 10 minutes at 72°C. The PCR products were then treated with *BsaI* enzyme (Thermo Fisher, USA) in the reaction buffer and separated on a 2% agarose gel to visualize the fragments. This method was adapted from Ziogas et al. [7].

## Supporting information

S1 Raw images (PDF)
