# Supplementary material for: A growing threat: Investigating the high incidence of benzimidazole fungicides resistance in Iranian Botrytis cinerea isolates
Source: PLoS One. 2023 Nov 21;18(11):e0294530. doi: 10.1371/journal.pone.0294530 (PMC10662737; doi:10.1371/journal.pone.0294530)
Supplement: S1 Raw images — (PDF) [file pone.0294530.s001.pdf]

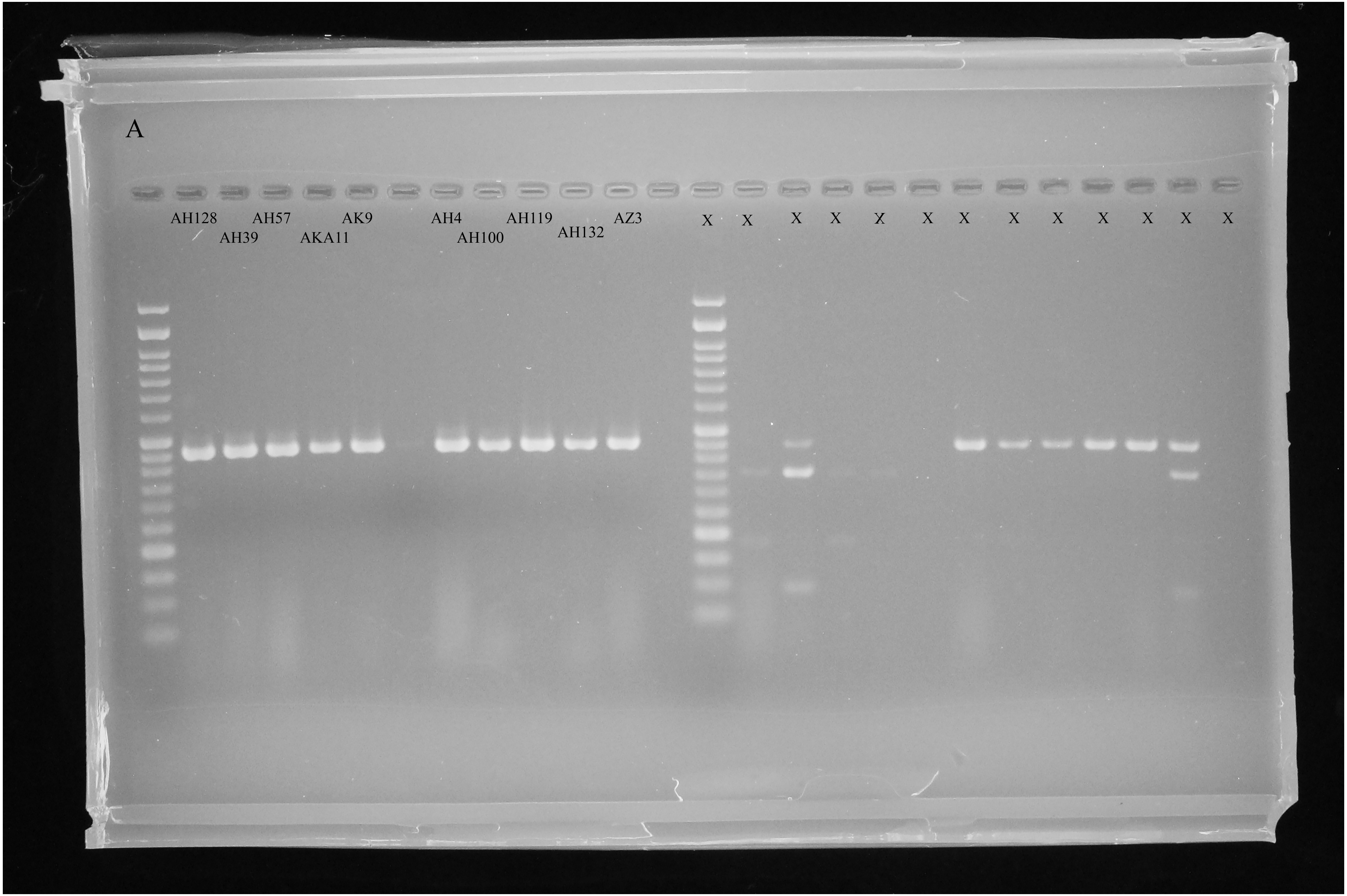
A
AH128
AH39
AH57
AKA11
AK9
AH4
AH100
AH119
AH132
AZ3
X
X
X
X
X
X
X
X
X
X
X
X
X

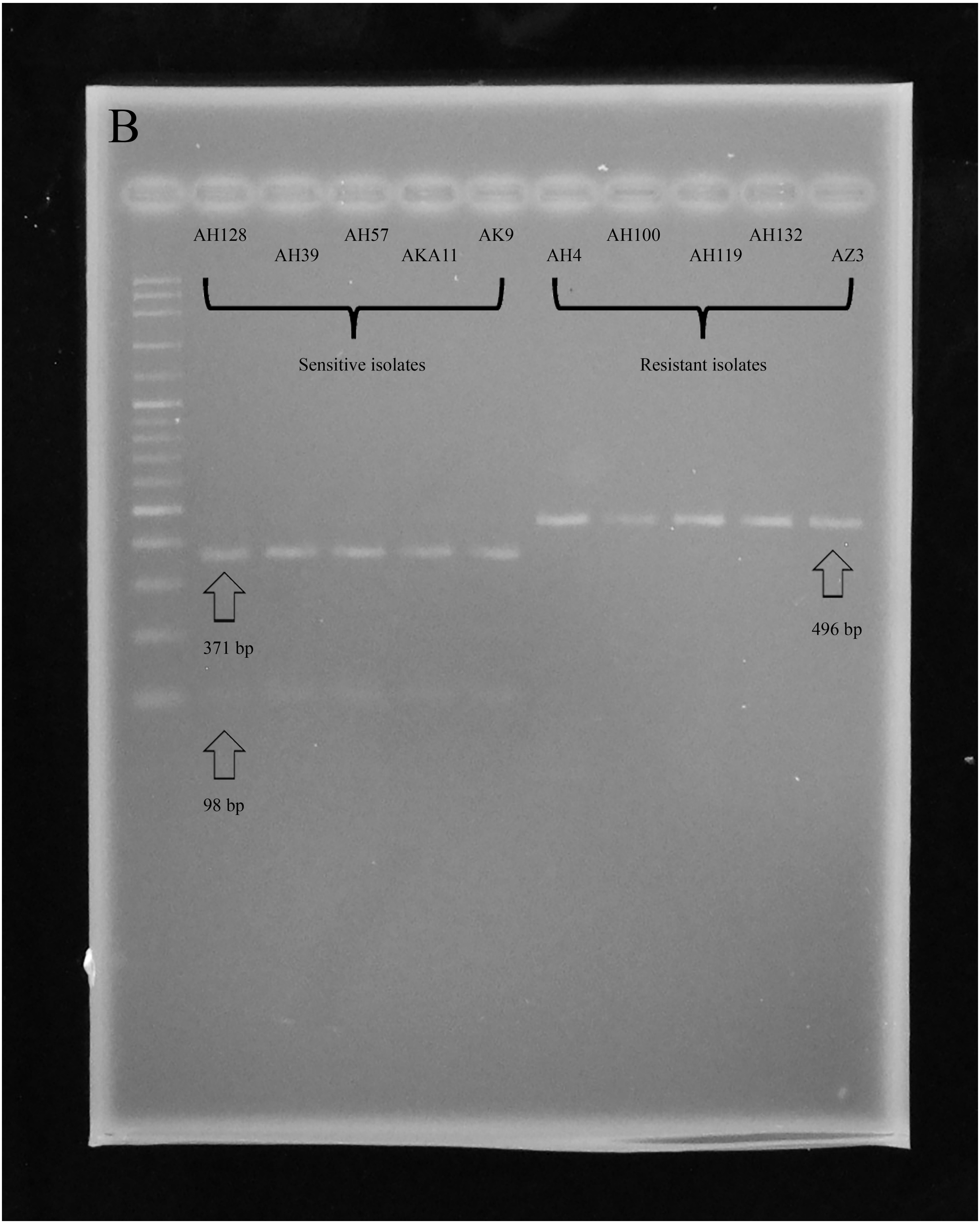
B
AH128
AH39
AH57
AKA11
AK9
AH4
AH100
AH119
AH132
AZ3
Sensitive isolates
Resistant isolates
371 bp
98 bp
496 bp

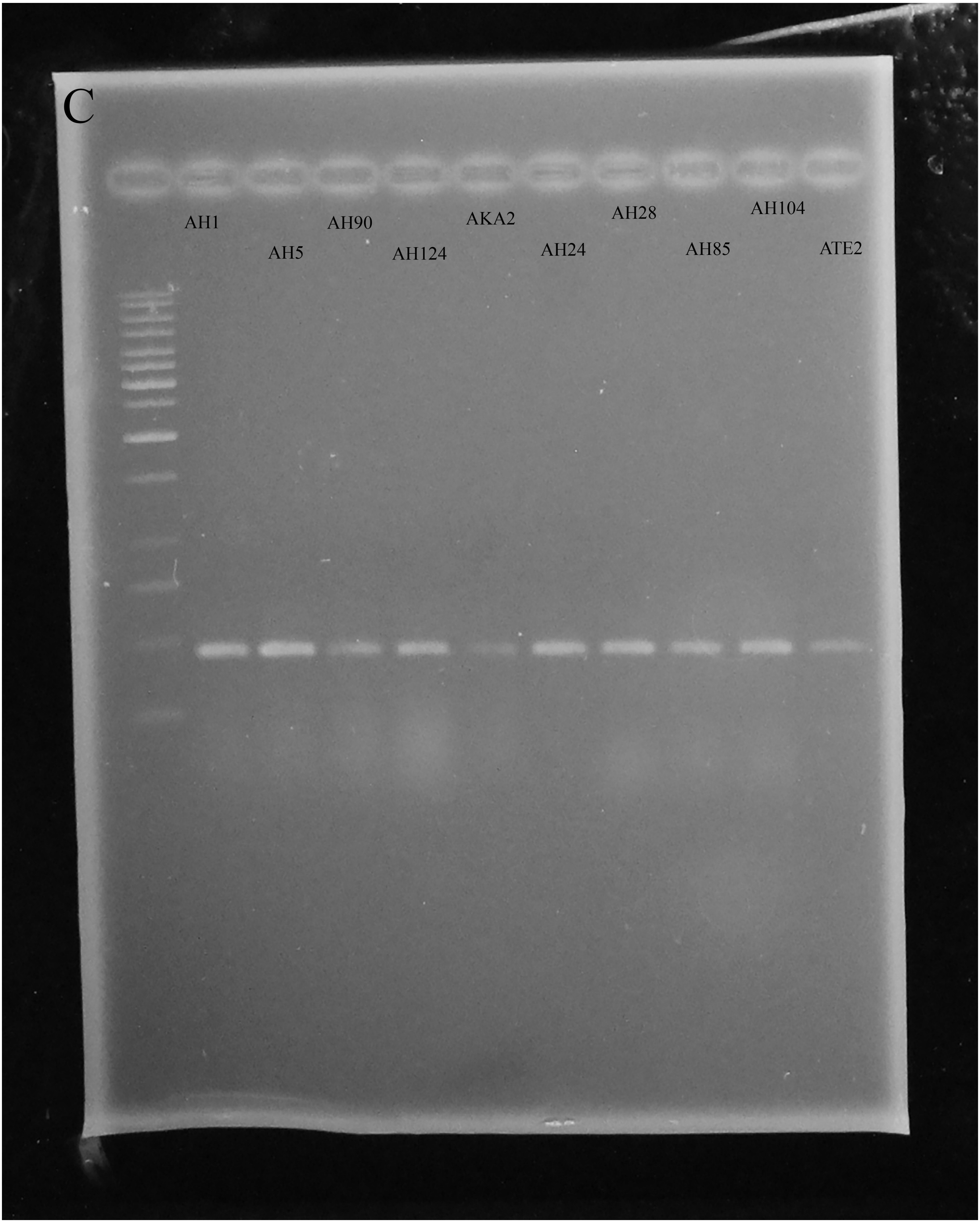
C
AH1
AH5
AH90
AH124
AKA2
AH24
AH28
AH85
AH104
ATE2

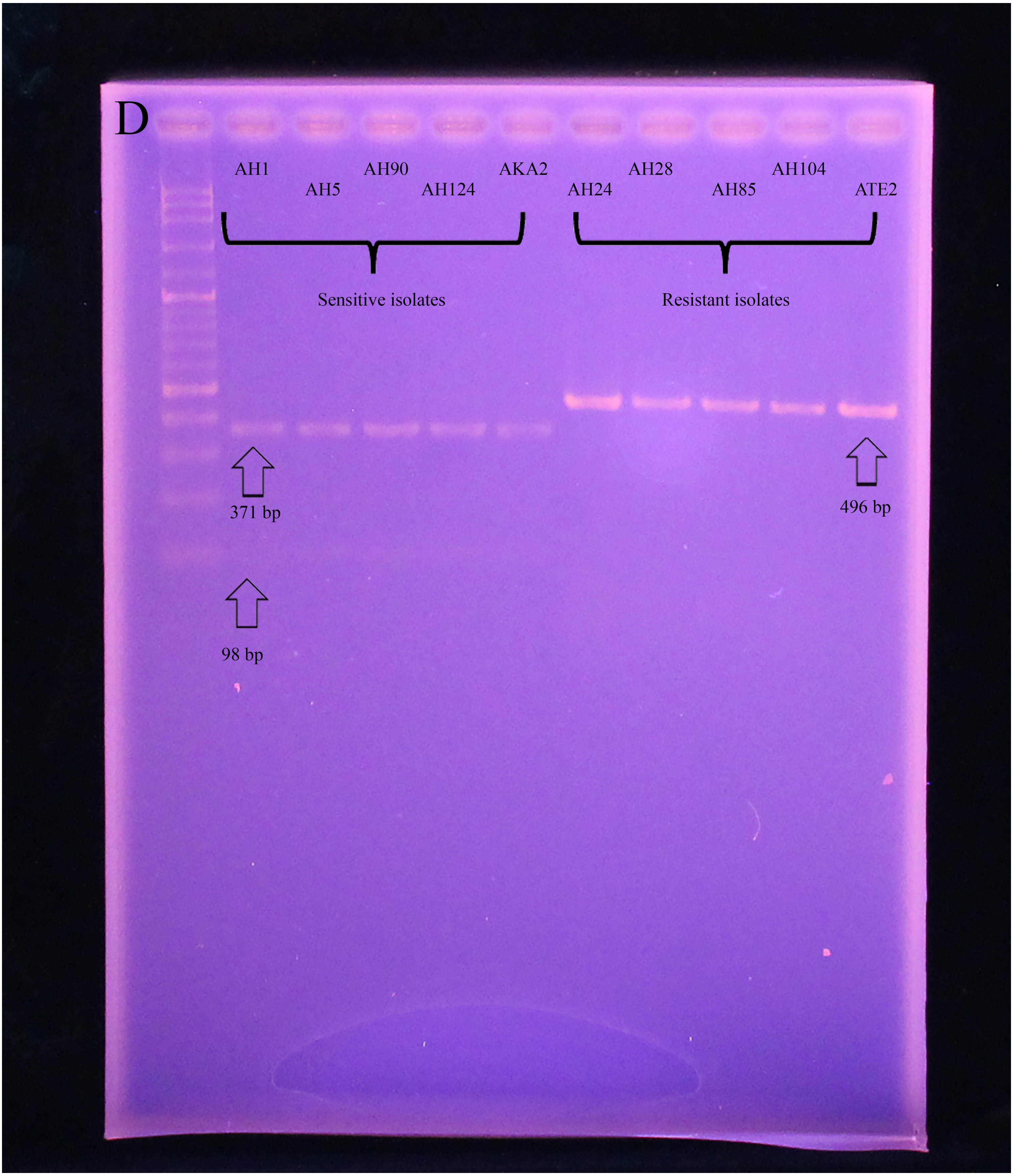
D
AH1
AH5
AH90
AH124
AKA2
AH24
AH28
AH85
AH104
ATE2
Sensitive isolates
Resistant isolates
371 bp
496 bp
98 bp
